# Predictors for Device-Detected Subclinical Atrial Fibrillation: An Up-to-Date Narrative Review

**DOI:** 10.3390/jcm15020578

**Published:** 2026-01-11

**Authors:** Traian Chiuariu, Larisa Anghel, Delia Melania Popa, Gavril-Silviu Bîrgoan, Șerban Daniel Fechet, Răzvan-Liviu Zanfirescu, Mircea Ovanez Balasanian, Radu Andy Sascău, Cristian Stătescu

**Affiliations:** 1 Internal Medicine Department, “Grigore T. Popa” University of Medicine and Pharmacy, 700503 Iași, Romania; traian.chiuariu@gmail.com (T.C.); deliamelaniapopa@gmail.com (D.M.P.); silviubirgoan@gmail.com (G.-S.B.); serban.fechet@umfiasi.ro (Ș.D.F.); ovanes718@yahoo.com (M.O.B.); radu.sascau@umfiasi.ro (R.A.S.); cristian.statescu@umfiasi.ro (C.S.); 2 Cardiology Department, Cardiovascular Diseases Institute “Prof. Dr. George I. M. Georgescu”, 700503 Iași, Romania; zanfirescu_razvan-liviu@d.umfiasi.ro; 3 Physiology Department, “Grigore T. Popa” University of Medicine and Pharmacy, 700503 Iași, Romania

**Keywords:** subclinical atrial fibrillation, atrial high-rate episodes, cardiac implantable electronic devices, predictive factors, atrial strain

## Abstract

**Background**: Device-detected subclinical atrial fibrillation (SCAF) and atrial high-rate episodes (AHRE) are increasingly recognized in patients with cardiac implantable electronic devices and through long-term rhythm monitoring. Although often asymptomatic, these episodes are associated with a higher risk of clinical atrial fibrillation (AF), stroke, and heart failure. **Aims**: This narrative review summarizes clinical, electrocardiographic, echocardiographic, and circulating biomarkers associated with the development and progression of device-detected SCAF/AHRE. **Methods**: We performed a comprehensive search of PubMed, Embase, and Scopus using combinations of the terms “subclinical atrial fibrillation”, “atrial high-rate episodes”, “device-detected AF”, “predictive factors”, “P-wave morphology”, “echocardiographic parameters”, “left atrial strain”, and “biological markers”. We included English-language-only studies of patients with cardiac implantable electronic devices or long-term monitoring and reporting incident SCAF/AHRE or AF as outcomes, published in the last 10 years. **Results**: Older age, high body mass index, heart failure, obstructive sleep apnea, and C_2_HEST score are consistently associated with SCAF. On-surface electrocardiogram (ECG) and device electrograms, prolonged and dispersed P-wave indices, low atrial sensing amplitude, and specific pacing configurations, particularly right ventricular apical pacing with wide QRS, predict incident and longer-lasting AHRE. Echocardiographic markers of atrial cardiomyopathy, including increased left atrial volume and impaired atrial strain, together with indices of left ventricular diastolic dysfunction, further refine risk. Among circulating biomarkers, galectin-3 and high-sensitivity C-reactive protein show the most reproducible associations with incident AHRE. **Conclusions**: A multiparametric approach combining clinical profile, ECG features, advanced echocardiography, and selected biomarkers may improve identification of patients at risk for device-detected SCAF. Further prospective studies are needed to define risk thresholds that justify intensified rhythm surveillance and early initiation of anticoagulation or rhythm control strategies, especially in AHRE shorter than 24 h.

## 1. Introduction

Cardiac implantable electronic devices (CIEDs) have significantly advanced long-term rhythm surveillance by continuously detecting, storing, and analyzing atrial electrical signals. Through these capabilities, a distinct clinical entity—device-detected subclinical atrial fibrillation (SCAF)—has been recognized. SCAF refers to asymptomatic atrial fibrillation (AF) episodes identified exclusively by continuous monitoring and is most frequently detected in patients with CIEDs equipped with an atrial lead, where a relevant proportion of atrial high-rate episodes (AHRE) represents true AF [1]. Despite widespread use of the term, there is no uniform definition of AHRE; different studies and device manufacturers apply atrial rate thresholds ranging from >170 beats/min [2], >175 beats/min [3], >190 beats/min [4,5] to >200 beats/min [3,6,7]. Likewise, the minimum episode duration varies, with commonly used cut-offs of 30 s [7,8], 5 min [1,9], or 6 min [4,5], most of which derive from investigations linking AHRE duration to thromboembolic risk.

Beyond implantable devices, SCAF may also be identified by consumer-grade wearable technologies, reflecting the increasing role of portable biosensors in arrhythmia detection [1]. Regardless of the monitoring tool, confirmatory evaluation by a qualified clinician is essential, as stored electrograms or wearable tracings may include false-positive signals [4]. AHRE classifications may also encompass atrial flutter or atrial tachycardia, while device-related artefacts—such as undersensing of low-amplitude atrial signals, functional undersensing occurring during blanking periods, or oversensing due to myopotentials, electromagnetic interference, or lead malfunction—can further complicate the diagnostic process [10].

Importantly, SCAF is not a harmless phenomenon. It has been established as an independent predictor of future clinically overt AF [5]. Among patients with known AF, silent episodes may represent up to 70% of total arrhythmic burden. Those who experience only subclinical episodes are at particular risk, as the absence of symptoms often delays the initiation of anticoagulation or rhythm-control therapy. Consequently, the first clinical manifestation may be stroke, systemic embolism, or heart failure (HF) [11]. Moreover, progression in SCAF duration correlates with an increased likelihood of heart-failure-related hospitalization [12].

**Figure 1:**
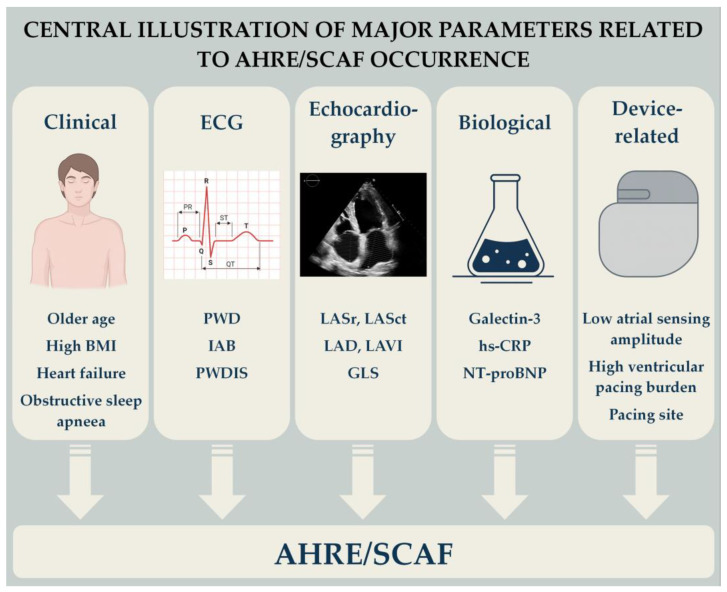
Central illustration of major parameters related to AHRE/SCAF occurrence. AHRE—atrial high-rate episode; BMI—body mass index; IAB—interatrial block; GLS—global longitudinal strain; hs-CRP—high-sensitivity C-reactive protein; LAD—left atrial diameter; LASr—Left atrial reservoir strain; LASct—left atrial conduit strain; LAVI—Left atrial volume index; NT-proBNP—N-terminal pro-B-type natriuretic peptide; PWD—P-wave duration; PWDIS—P-wave dispersion; SCAF—subclinical atrial fibrillation.

The aim of this review is to integrate evidence on AHRE predictors spanning clinical, electrocardiographic, echocardiographic, and biological measures—together with additional device and lead characteristics—to build a pragmatic framework for risk assessment in CIED populations (Figure 1).

## 2. Materials and Methods

This narrative review integrates the most relevant evidence published over the past decade regarding the clinical, electrocardiographic, echocardiographic and biological markers associated with the development of device-detected SCAF. The objective was to compile and critically appraise data from observational studies, randomized trials, device registries, and imaging-based investigations that report predictors of SCAF or atrial high-rate episodes in populations undergoing continuous rhythm monitoring.

A comprehensive literature search was performed across PubMed, Embase, and Scopus databases. The search strategy combined Medical Subject Headings (MeSH) and free-text terms related to subclinical atrial arrhythmias and their predictors. The primary keywords and keyword combinations included “subclinical atrial fibrillation”, “atrial high-rate episodes”, “device-detected AF”, “predictive factors”, “P-wave morphology”, “echocardiographic parameters”, “left atrial strain”, and “biological markers”. Boolean operators (AND/OR) were applied to refine and broaden the search where appropriate.

Studies were eligible for inclusion if they met the following criteria:

1. Included adult patients monitored by cardiac implantable electronic devices, implantable loop recorders, or long-term external monitoring systems;
2. Reported incident SCAF/AHRE or progression of arrhythmic burden as an outcome;
3. Evaluated clinical, ECG, echocardiographic, or biomarker-based predictors;
4. Provided extractable quantitative or qualitative data relevant to SCAF risk stratification.

Both prospective and retrospective studies were considered. Reference lists of key articles and recent guidelines were also screened to identify additional relevant publications. No restrictions were applied regarding geographic location, but only studies published in English in the past 10 years were included.

The selection and synthesis of the evidence adhered to recognized standards for narrative reviews, emphasizing methodological transparency and critical interpretation rather than formal meta-analytic pooling. The aim was to provide a comprehensive and integrated understanding of the factors that predispose to SCAF in contemporary device-monitored populations.

## 3. Results

### 3.1. Clinical Factors

Clinical features remain fundamental to risk assessment in patients with CIEDs. Factors such as advanced age, the presence of cardiovascular comorbidities, and traditional risk scores have consistently been associated with a higher probability of developing SCAF. Over the past decade, growing evidence has highlighted these variables not only as independent predictors but also as essential anchors upon which more advanced diagnostic parameters can be layered to improve early identification of at-risk individuals.

Advancing age is the most consistent clinical determinant of device-detected atrial arrhythmias. In a real-world cohort of CIED carriers, age remained independently associated with the first occurrence of AHRE after multivariable adjustment, alongside prior AF and inflammatory indices [13]. Beyond onset, age also tracks disease trajectory; in a prospective device-monitoring programme, older age independently predicted progression from brief subclinical AF to sustained episodes (>24 h) or clinical AF, a transition that carries higher thromboembolic relevance [12].

Excess body weight appears to act as a dose-dependent substrate for device-detected atrial arrhythmias. In a pacemaker/implantable cardiac defibrillator (ICD) cohort, patients with AHRE had higher body mass index (BMI) than those without, and after multivariable adjustment, class I obesity (BMI 30–35 kg/m^2^) remained independently associated with AHRE [14]. In the ASSERT substudy, BMI was an independent predictor of SCAF progression from brief episodes (>6 min) to SCAF > 24 h or clinical AF, with each 10 kg/m^2^ increment conferring an ≈80% higher risk of progression (hazard ratio [HR] 1.83, 95% CI 1.14–2.94) [12]. Mechanistically, obesity promotes left-atrial enlargement, epicardial adipose deposition, low-grade inflammation, and renin–angiotensin–aldosterone activation, all of which favour atrial ectopy and re-entry [15]. Taken together, these data support elevated BMI as a modifiable upstream determinant of AHRE burden in CIED recipients.

Hypertension is a common comorbidity in CIED recipients, but its independent association with incident AHRE is inconsistent. Hypertension appears to act more as a substrate amplifier—partly through its link to atrial enlargement—than as a standalone trigger of AHRE in CIED cohorts, with several cohorts showing significance on univariate analysis but loss of significance after multivariable adjustment [16,17,18].

HF and SCAF show a bidirectional relationship in CIED populations. In a systematic review of 54 CIED cohorts (>72,000 patients), SCAF prevalence was 28.1%, and chronic HF was independently associated with SCAF presence (odds ratio [OR] 1.39, 95% CI 1.06–1.83), alongside older age, hypertension, and prior stroke/transient ischemic attack (TIA)(h [19]. In the West Birmingham Atrial Fibrillation Project (500 CIED patients without prior AF), age and systolic heart failure were independent predictors of sustained AHREs (>24 h), and these episodes were associated with higher all-cause mortality and ischemic stroke [17]. Conversely, prospective CIED studies show that new-onset AHRE and higher AHRE burden independently predict HF worsening or HF hospitalization, both in unselected CIED cohorts and in cardiac resynchronization therapy defibrillator (CRT-D) recipients with reduced left ventricular ejection fraction (LVEF)—risk for HF hospitalization with SCAF increased by ≈4.5 in CRT-D patients [20,21].

Diabetes mellitus is frequent in CIED recipients, but its relationship with AHRE incidence appears modest and largely confounded. In a contemporary dual-chamber pacemaker cohort, the prevalence of type 2 diabetes was similar in patients with and without AHRE and did not emerge as an independent predictor once prior atrial arrhythmias and pacing variables were included in the multivariable model [22]. In a separate cohort of HF with reduced ejection fraction patients with CIEDs, classical risk factors including diabetes were common, but AHRE occurrence was independently predicted by lower left-ventricular ejection fraction, larger left-atrial diameter, and higher uric acid levels, again suggesting that structural and biochemical markers outweigh diabetes itself in this setting [23]. Likewise, in a large ICD/CRT-D remote-monitoring registry, diabetes lost its association with new-onset AHRE after adjustment and propensity matching, although diabetic patients who did develop AHRE had a markedly higher subsequent mortality than diabetic patients without AHRE [24]. Overall, diabetes seems to function more as a prognostic modifier once device-detected AF is present than as a robust independent predictor of AHRE onset.

Thyroid dysfunction has been only sparsely evaluated as a predictor of AHRE in CIED cohorts, and the available data suggest a weak but biologically plausible signal. In the West Birmingham AF Project, hyperthyroidism was rare (1.2% overall) but more frequent in patients who developed sustained AHRE > 24 h than in those without sustained AHRE (4.5% vs. 0.9%; *p* = 0.033), although the small numbers prevented demonstration of an independent effect after multivariable adjustment [17].

Obstructive sleep apnoea (OSA) is common but often under-recognized in CIED recipients, and device-based monitoring has clarified its relationship with atrial arrhythmia burden. In pacemaker patients with embedded respiratory algorithms, severe sleep apnoea—defined by a pacemaker-derived respiratory disturbance index ≥ 30 events/hour—was detected in about 60% of individuals and independently doubled to almost tripled the risk of device-detected AF episodes ≥ 6 h/day during follow-up, even after adjustment for prior AF and conventional risk factors [25]. Pacemaker-based studies also show that OSA is highly prevalent and fluctuates over time in this population, reinforcing the concept of OSA burden as a dynamic atrial trigger rather than a static comorbidity [25]. More recently, in 411 HF patients with ICDs equipped with multiparametric sensors, La Greca et al. demonstrated that ICD-detected severe sleep apnoea (respiratory disturbance index ≥ 30 episodes/hour) was independently associated with having a daily AHRE burden ≥ 5 min, alongside indices of decompensated HF and reduced physical activity, although it did not predict very-long-burden AHRE ≥ 23 h/day [26]. Complementing these findings, a retrospective cohort reported a clear dose–response relationship between device-detected OSA severity and atrial arrhythmia duration; for each 1-unit increase in a remote-derived apnoea-hypopnoea index, daily AHRE burden increased by ~10 s, and patients with mild, moderate, or severe OSA had 2.8–3.0 additional hours of atrial arrhythmia per day compared with those without OSA [27]. Mechanistically, OSA promotes atrial ectopy and remodelling through intermittent hypoxia, large negative intrathoracic pressure swings, sympathetic surges, and inflammation/fibrosis, all of which have been linked to AF initiation and maintenance in both experimental work and clinical mapping studies [28].

It is also noteworthy that conventional clinical risk scores (APPLE, ALARMEc, CHA_2_DS, CHA_2_DS_2_-VASc) have exhibited limited discriminatory capacity for predicting the occurrence of device-detected events [13]. The key relevance of AHRE is their high risk of progressing to clinical AF. Chen et al. [29] demonstrated that AHRE ≥ 6 min, AHRE ≥ 24 h, and elevated HAT_2_CH_2_ score (hypertension, age > 75 years, prior stroke or TIA, chronic obstructive pulmonary disease, and heart failure) independently predict new-onset clinical AF in previously unaffected individuals. Furthermore, the C_2_HEST multiparametric risk score (Coronary artery disease or COPD, Hypertension, Elderly age ≥ 75 years, Systolic heart failure, Thyroid disease) showed a ≈40% per-point increase in the risk of sustained AHRE > 24 h [17].

### 3.2. Electrocardiographic Factors

Building upon the clinical profile, electrocardiographic parameters offer essential insight into the atrial electrical remodelling that precedes the development of SCAF. Surface electrocardiogram (ECG) markers frequently uncover subtle conduction disturbances and arrhythmogenic patterns that may not yet manifest clinically. These electrical alterations complement traditional clinical predictors and help identify patients at increased risk. Among these, P-wave indices (PWIs) are particularly informative, as they reflect atrial conduction properties and serve as indirect markers of structural remodelling. Altered atrial activation patterns captured through PWI analysis have been associated with atrial architectural changes and a higher likelihood of ischemic stroke [30].

Because mechanical atrial function follows electrical depolarization, ECG changes may also provide indirect evidence of impaired cardiac mechanics. Tekkeșin et al. [31] explored this relationship in a cross-sectional study examining diastolic ECG parameters in relation to SCAF occurrence. Given the established link between diastolic dysfunction and atrial mechanical impairment, which may foster a pro-arrhythmic substrate, the authors reported significantly shorter Tend-P (RR–PQ–QT) and Tend-Q (RR–QT) intervals in patients with AHRE, along with a longer PQ interval compared with controls. A PQ interval > 151 ms demonstrated excellent specificity (100%) but only moderate sensitivity (58.8%) for predicting AHRE, limiting its utility as a standalone screening tool. PQ prolongation, consistent with earlier findings such as those from the Framingham Heart Study, suggests delayed atrial conduction and possible structural remodelling. AHRE patients in this study also showed longer QTc intervals and a markedly lower diastolic ECG index [Tend-P/(PQ × age)].

Additional insight comes from the LOOP study, a large randomized trial assessing whether systematic screening for subclinical AF using implantable loop recorders reduces stroke risk. In a subanalysis, Xing et al. [32] evaluated various 12-lead ECG parameters—PR interval, P-wave duration (PWD), P-wave voltage in lead I (PWV), P-wave axis (PWA), P-wave terminal force in V1 (PTF), interatrial block (IAB), QTc interval, QRS duration, and QRS-T angle—to determine their association with AF detection. Abnormal PR intervals (<120 or >200 ms), prolonged PWD (>120 ms), low PWV (<100 µV), abnormal PWA (<0° or >75°), IAB (PWD ≥120 ms with biphasic P waves in the inferior leads), and prolonged QTc (>450 ms) were all linked to higher AF detection rates. The median time to the first AF episode was 1.01 years after device implantation. Although several ECG variables were associated with AF occurrence, only prolonged PWD and the presence of IAB independently predicted episodes lasting > 24 h—durations known to confer increased thromboembolic risk [5]. Notably, longer QRS duration was associated with a greater number of short-lasting AF episodes, contributing to increased total AF burden despite the absence of sustained episodes, underlining the need for further studies to clarify the prognostic relevance of cumulative AF burden [2,3].

Campal et al. [33] evaluated whether P-wave duration recorded during sinus rhythm and atrial pacing, both before and after CIED implantation, could predict subsequent AHRE. Given that pacing typically produces a longer P-wave compared to sinus rhythm, the authors demonstrated that a paced P-wave duration > 160 ms was significantly associated with AH-RE—particularly with episodes exceeding 24 h.

Karakayali et al. [34] further analyzed PWIs in patients with HF with reduced ejection fraction and found that P-wave peak time (PWPT) in lead V1 (PWPTV1) and lead II (PWPTD2) were significantly prolonged in individuals with AHRE. The proposed cut-off values were 37 ms for PWPTV1 and 45 ms for PWPTD2.

Zhu et al. [35] conducted a retrospective assessment of patients after pacemaker implantation and introduced the morphology-voltage-P-wave (MVP) score, integrating PWD, IAB, and PWV in lead I. High cumulative atrial and ventricular pacing, elevated MVP score during pacing, prolonged PW-peak times (sinus and paced), and increased sinus PWD were all associated with AHRE. Among these, high cumulative ventricular pacing emerged as an independent predictor, while MVP score and paced PWPT predicted prolonged AHRE (>24 h). Interestingly, partial and advanced IAB did not correlate with AHRE in this cohort, a finding consistent with the results of Valdez Baez et al. [22].

Another parameter is the P wave dispersion (PWDIS), defined as the difference between the maximum and the minimum P-wave durations detected on the body surface 12-lead ECG. Nishinarita et al. discovered that PWDIS is an independent predictor of new-onset AHRE, with a cut-off value of 48 ms [36].

### 3.3. Echocardiographic Factors

Structural and functional cardiac imaging provides an essential layer of information in assessing the risk of device-detected SCAF. Conventional echocardiographic measurements, together with advanced modalities such as left atrial (LA) strain imaging, help characterize the atrial substrate that predisposes to arrhythmia. Atrial mechanical dysfunction and fibrosis—frequently detected through these imaging markers—play a central role in the initiation and maintenance of SCAF. When combined with clinical and electrocardiographic parameters, echocardiographic findings substantially improve early identification of patients at higher risk

#### 3.3.1. Left Atrial Strain

Myocardial strain quantifies the percentage change in myocardial length using frame-to-frame tracking of acoustic “speckles” on two-dimensional echocardiography; myocardial shortening is expressed as a negative value, whereas lengthening is reported as a positive value [37]. The left atrium exhibits three mechanical phases throughout the cardiac cycle—reservoir during ventricular systole, conduit during early diastole, and booster-pump during atrial systole—and speckle-tracking echocardiography provides corresponding strain indices for each of these phases [38].

Reduced LA strain reflects impaired atrial compliance and fibrosis. Lower strain values correlate with late gadolinium enhancement (LGE) on cardiac magnetic resonance (CMR) and with histological markers of fibrosis, supporting the concept of LA strain as a sensitive indicator of atrial remodelling and arrhythmogenic vulnerability [39,40].

Multiple prospective studies underscore the value of LA strain in predicting subclinical atrial arrhythmias. In the LOOP echocardiographic substudy (*n* = 956; median follow-up 35 months), each 1% reduction in reservoir strain (LASr) independently predicted SCAF (HR 1.04; 95% CI 1.02–1.05), while LASr < 33% identified patients at increased risk even in the absence of LA enlargement or diastolic dysfunction; similar findings were observed for conduit strain (LASct) [41]. Among CIED recipients (*n* = 105), lower LASct independently correlated with AHRE (adjusted OR ≈ 1.18 per 1% decrease), with a cut-off of −4.125% offering high specificity (≈88%) [42]. Additional work has shown that worsening LA strain is associated with both the occurrence and the duration of AHRE, reinforcing atrial myopathy as the underlying substrate for these device-detected events [42,43].

The predictive capacity of atrial strain extends beyond CIED cohorts. In individuals with cryptogenic stroke or embolic stroke of undetermined source, reduced LA or left atrial appendage strain predicted subsequent SCAF during continuous monitoring, highlighting that structural substrate can be detected even in the absence of chamber enlargement [44].

Beyond echocardiography, emerging cardiac computed tomography (CT) techniques now permit automated 3D LA strain quantification through deep-learning algorithms. In exploratory analyses, patients with AHRE > 6 min demonstrated significantly lower global strain and LA emptying fraction compared to those with AHRE ≤ 6 min, suggesting that atrial dysfunction can also be characterized through CT-derived metrics [45].

#### 3.3.2. Left Atrial Size

Left atrial enlargement represents the macroscopic footprint of chronic atrial remodelling driven by sustained elevations in left ventricular filling pressures and cumulative pressure or volume load [46]. Echocardiography measures left atrium (LA) size primarily through LA volume—preferably indexed to body surface area (LAVI)—or through linear diameters. Contemporary guidelines endorse biplane LAVI at end-systole as the most accurate echocardiographic parameter because it better reflects true atrial remodelling than single-dimension diameters [47].

Accurate assessment is complicated by the atrium’s asymmetric geometry, variable wall thickness, and the influence of structures such as the pulmonary veins and left atrial appendage. Although antero-posterior diameter is commonly used due to its reproducibility, it insufficiently captures asymmetric remodelling [46,48]. Furthermore, transthoracic echocardiography tends to underestimate LA volume compared with CMR, with studies demonstrating systematic negative bias and risk of misclassification when the two modalities are compared [49].

Across device-monitored populations, larger LA size consistently associates with AHRE. A recent meta-analysis of patients without prior AF demonstrated that those with AHRE had significantly greater LA diameter and higher LAVI [50]. In a single-centre cohort of CIED recipients, LA diameter emerged as the only echocardiographic parameter consistently predicting AHRE ≥ 6 min, ≥6 h, and ≥24 h during a median follow-up of 29 months, underscoring its practical value for routine risk assessment [51].

Similarly, in a multicentre pacemaker registry, an LA diameter (LAD) > 41 mm identified individuals at elevated risk for prolonged AHRE. LAD > 41 mm independently predicted AHRE > 6 min (OR 2.08; 95% CI 1.25–3.45) and AHRE > 6 h (OR 1.96; 95% CI 1.00–3.85), with most events detected within the first six months after implantation [16]. In another cohort of patients with sick sinus syndrome (n = 118), each 1 mm increase in LAD was associated with a 10% higher hazard of subsequent AHRE (HR 1.10; 95% CI 1.04–1.18) [52].

#### 3.3.3. Mitral Doppler Profile and Left Ventricular Diastolic Dysfunction

Elevated left ventricular (LV) filling pressures, caused by impaired relaxation or increased myocardial stiffness, result in atrial stretch and progressive remodelling that facilitate fibrosis formation [46,53]. This structural alteration contributes directly to the arrhythmogenic substrate underlying AF [54,55]. Standard echocardiographic assessment of diastolic function relies on transmitral Doppler indices (E, A, E/A, deceleration time) and tissue Doppler e′ velocities, with the E/e′ ratio serving as a surrogate for LV filling pressure. Given the load-dependence of these parameters, expert recommendations advocate for a multiparametric algorithm that includes transmitral inflow, tissue Doppler, LA structure/function, and, when available, LA strain [46,56].

In a cohort of 203 CIED recipients, the left atrial coupling index (LACI)—calculated as the ratio of LAVI to the medial a′ wave—proved superior to traditional Doppler markers in predicting AHRE. LACI remained an independent predictor of AHRE (OR 1.752; 95% CI 1.356–2.263) and outperformed LAVI on ROC analysis [57]. In patients with cryptogenic stroke monitored by implantable loop recorders, those who developed AF displayed higher transmitral E velocity and elevated E/e′ ratios compared with AF-free individuals, indicating impaired diastolic function [58]. Conversely, in a dual-chamber pacemaker cohort, neither E/e′ nor E/A was associated with AHRE on univariate analysis, indicating possible population-specific variability [18].

Additional evidence supports the role of diastolic myocardial stiffness. Diastolic wall strain (DWS), a non-invasive marker of LV stiffness, was independently linked to AHRE in patients without prior AF. Lower DWS—indicating increased stiffness—was associated with prevalent AHRE, suggesting that impaired LV compliance contributes to atrial remodelling and lowers the threshold for subclinical atrial arrhythmias [59].

#### 3.3.4. Left Ventricular Systolic Function

Left ventricular systolic performance also appears to influence susceptibility to AHRE. Several cohorts report lower LVEF among AHRE-positive patients when compared with AHRE-negative individuals, often in parallel with larger LA size [3]. However, findings are not uniform; some studies have observed no significant difference in LVEF between groups [60]. These discrepancies suggest that LVEF alone may lack sensitivity for predicting AHRE and that more refined systolic markers may provide superior risk stratification.

Supporting this view, a prospective cohort of 145 patients with newly implanted dual-chamber pacemakers found that impaired LV global longitudinal strain (GLS)—a sensitive indicator of subclinical systolic dysfunction—independently predicted AHRE (HR 0.92 per 1% decrease; 95% CI 0.84–0.99) [61].

Taken together, combining LVEF with GLS provides a more comprehensive assessment of LV systolic performance. GLS consistently captures subtle myocardial dysfunction associated with AHRE risk more closely than LVEF, offering added prognostic value.

### 3.4. Biological Factors

Circulating biomarkers offer insight into the molecular mechanisms underlying SCAF, reflecting key processes such as atrial fibrosis, inflammation, and structural remodelling. Biomarkers have gained attention for their potential to link biochemical pathways with both functional and electrical atrial abnormalities. Their correlations with echocardiographic and electrocardiographic indices suggest that integrating biomarker data with imaging and ECG parameters may provide a more refined assessment of individual arrhythmic vulnerability.

Galectin-3 has emerged as a promising candidate linking biological activity to functional and electrical atrial alterations. In patients treated with cardiac resynchronization therapy (CRT), elevated Galectin-3 levels measured in the coronary sinus have been identified as strong independent predictors of incident AHRE, with higher concentrations also correlating with increased AHRE burden. These findings strengthen the association between profibrotic signalling and the development of subclinical atrial arrhythmias [62]. However, results in other device-monitored populations are less consistent. In dual-chamber pacemaker recipients, individuals who developed AHRE exhibited higher baseline Galectin-3 levels, but the marker’s predictive value diminished after multivariable adjustment, with left atrial volume index emerging as the only independent predictor of AHRE in that cohort [63].

Inflammatory activity has also been implicated in the pathogenesis of AHRE. In a prospective study of pacemaker recipients with preserved ejection fraction, high-sensitivity C-reactive protein (hs-CRP), a more sensitive assay for C-reactive protein (CRP), was confirmed as an independent predictor of AHRE lasting ≥ 6 min. A refined threshold of hs-CRP > 0.525 mg/L improved discrimination and showed a clear association with higher AHRE burden. Notably, other commonly measured cardiac biomarkers—including N-terminal pro–B-type natriuretic peptide (NT-proBNP), high-sensitivity troponin T, and D-dimer—did not demonstrate predictive value in the same analysis, highlighting a divergence between subclinical device-detected arrhythmias and overt clinical AF where these markers typically perform well [64]. This pattern supports a pathophysiological model in which chronic atrial inflammatory remodelling, rather than acute hemodynamic stress or myocyte necrosis, is the dominant substrate for device-detected episodes. Complementary evidence comes from a real-world multicentre cohort of CIED carriers in which elevated CRP and white-cell count—alongside age and prior AF—were independent predictors of AHRE onset [13].

Exploratory device-based evidence also links monocyte-to-HDL ratio (MHR)—a composite marker of inflammation/oxidative stress—to early AHRE after dual-chamber pacemaker implantation; in a single-centre study (n = 203), higher baseline MHR was associated with AHRE at 6 months, retaining independent predictive value on multivariable analysis [65]. A later analysis reported similar findings within one year from the moment of implant, supporting external consistency of MHR as a prognostic signal in CIED cohorts [66].

Furthermore, the assessment of myocardial stretch and injury biomarkers holds significant relevance for AHRE prediction. Signals from myocardial wall stress and cardiomyocyte injury—principally NT-proBNP and high-sensitivity troponin—are heterogeneous in device-monitored cohorts and appear to depend on episode duration and underlying substrate. In a dual-chamber pacemaker case–control study with continuous device surveillance, NT-proBNP was higher in patients with recent AHRE ≥ 6 min and paroxysmal AF than in pacemaker controls without AHRE, with an NT-proBNP threshold > 150 pg/mL conferring a markedly increased odds of AHRE [67]. In contrast, prospective device screening with implantable loop recorders (ILRs) suggests that natriuretic peptides and troponin T have their strongest association with longer subclinical AF episodes; in the LOOP program, baseline NT-proBNP and troponin T independently predicted AF detection and improved prediction of episodes ≥ 24 h when added to age/sex/comorbidity models, implying that stretch/injury signals track higher AF burden rather than short, intermittent runs [68].

Interpretation of troponin in CIED populations is further complicated by procedure and pacing-related release that is unrelated to atrial arrhythmogenesis. The rise in troponin I after de novo pacemaker implantation correlates with lead fixation and procedural complexity, indicating true but localized myocardial injury unrelated to subsequent AHRE risk [69]. Experimental and catheterization-laboratory data also show that rapid atrial pacing can provoke detectable troponin T release through transient supply–demand mismatch, underscoring limited specificity for arrhythmic prediction [70].

Taken together, device-anchored evidence indicates that NT-proBNP (and possibly troponin) may enrich for more sustained subclinical AF burden in ILR cohorts, whereas prediction of short-duration AHRE in pacemaker recipients is inconsistent and readily attenuated by structural confounding. These observations support a pragmatic approach in which stretch/injury biomarkers are integrated with atrial structure and function, rather than used alone, when modelling AHRE risk in CIED-monitored patients.

### 3.5. Device-Related Factors

Beyond the clinical, electrical, and structural predictors, device- and pacing-related characteristics also provide important prognostic information in CIED carriers. Parameters obtained from routine device interrogation, device-based diagnostic algorithms, and detailed assessment of pacing site have all been linked with subsequent SCAF/AHRE burden. These device-centred metrics complement the previously discussed risk domains and help build a more granular, multiparametric framework for early detection and individualized management.

For instance, abnormalities in device-derived atrial signals consistently track subsequent AHRE. Lower atrial sensing amplitudes—typically below 2.45 mV or 1.5 mV—identify patients at higher risk for both incident episodes and progression, with longitudinal data showing a gradual fall in amplitude during the year before the first detected event [71,72].

Another significant factor related to AHRE development was the cumulative percentage of ventricular pacing ≥ 50% [33]. Pacing strategy has become a key area of interest. Compared with conventional right ventricular pacing (RVP), left bundle branch area pacing (LBBAP) has been associated with a lower incidence of device-detected AHRE and superior preservation of systolic function in patients with atrioventricular block [73,74]. In a propensity-matched cohort of individuals without prior AF, LBBAP significantly reduced the hazard of new-onset AHRE (HR 0.274; 95% CI 0.113–0.692) and maintained left ventricular ejection fraction (LVEF) at 1 year, whereas RVP was associated with a decline in systolic function [75]. Moreover, comparisons between conduction-system pacing modalities suggest that both His-bundle pacing and LBBAP exhibit similar rates of AHRE on medium-term follow-up, implying that the primary benefit arises from minimizing ventricular dyssynchrony rather than from a specific pacing target [76].

Building on early observational findings from ASSERT, atrial high-rate episodes and subclinical AF have been consistently associated with subsequent clinically overt AF and a higher thromboembolic risk, with risk increasing in relation to episode duration and cumulative arrhythmia burden [5]. Importantly, time-dependent analyses from ASSERT suggest that the excess thromboembolic risk is largely driven by longer SCAF episodes, with the most robust signal observed when episode duration exceeds 24 h, whereas shorter episodes (<24 h) show a weaker and less consistent association. These observations have practical relevance because they imply that AHRE is not a homogeneous entity and that “one-size-fits-all” anticoagulation cannot be assumed to provide net benefit in all patients without ECG-documented AF [77]. Recent randomized trials further support a more selective approach. In ARTESiA (apixaban vs. aspirin), a high-risk population (mean CHA_2_DS_2_-VASc score of 3.9 ± 1.1) with predominantly short SCAF episodes (median longest episode of 1–2 h) experienced a reduction in stroke/systemic embolism with apixaban, but at the cost of increased major bleeding—highlighting a trade-off that requires individualized decision-making [78]. In NOAH-AFNET 6 (edoxaban vs. placebo) among patients with AHRE without ECG-confirmed AF (median CHA_2_DS_2_-VASc score of 4; median longest AHRE of 2.8 h), edoxaban did not significantly reduce the composite of cardiovascular death, stroke, or systemic embolism and increased major bleeding; notably, overall stroke rates were low [79]. Taken together, these data indicate that routine anticoagulation for AHRE/SCAF—especially when episodes are short—should not be automatically extrapolated from clinical AF, and that the net clinical benefit likely depends on (i) episode duration/burden, (ii) baseline stroke risk, and (iii) evidence of atrial cardiomyopathy/remodelling.

Therefore, beyond traditional clinical scores, there is a clear need to refine decision thresholds using a multiparametric framework that integrates AHRE duration and burden with markers of atrial substrate (e.g., left atrial mechanics/strain, atrial size and fibrosis surrogates, ECG indices, and biomarkers). Such an approach may better identify the subgroup most likely to derive net benefit from anticoagulation while avoiding unnecessary bleeding risk in patients with low absolute event rates [25,26,27].

Regarding ECG-based markers, refinements in the quantification of atrial electrical substrate—including interatrial block and advanced P-wave indices (including paced P-wave measures and derived scores such as the MVP score)—have been proposed to improve prediction of incident and/or prolonged AHRE (e.g., >24 h) beyond conventional ECG descriptors [5,32,33,35], alongside the introduction of specific indices such as PWDIS with suggested cut-offs [36]. Regarding echocardiography and cardiac imaging parameters, the focus has increasingly moved from “size-only” assessment toward atrial (and global myocardial) function, with left atrial strain (LASr/LASct) emerging as a sensitive marker of atrial cardiomyopathy and proposed thresholds for higher AHRE/SCAF risk even in the absence of overt atrial dilatation [41,42], and with LV global longitudinal strain (GLS) being explored as an additional functional marker associated with AHRE [61]. Regarding circulating biomarkers, interest has increasingly shifted toward upstream remodelling pathways underlying disease progression—particularly fibrosis and inflammation—supported by associations between galectin-3 and AHRE incidence and/or burden [62,63], and by inflammatory markers such as hs-CRP in selected cohorts [13,64], while natriuretic peptides appear especially informative for longer SCAF episodes (e.g., ≥24 h) [67,68]. Finally, within the device/pacing domain, emerging “device-analytics” signals such as atrial sensing amplitude and its temporal trends have been investigated as early markers of atrial electrical remodelling and impending AHRE [71,72], and pacing strategy innovations—especially conduction system pacing (including LBBAP)—have been linked to lower AHRE incidence and more favourable functional profiles compared with conventional pacing approaches [73,74,75,76].

Taken together, all these clinical, electrocardiographic, echocardiographic, biomarker, and device-related parameters form a multiparametric framework for AHRE/SCAF risk stratification. The main predictors across these domains are summarized in Table 1.

**Figure 2:**
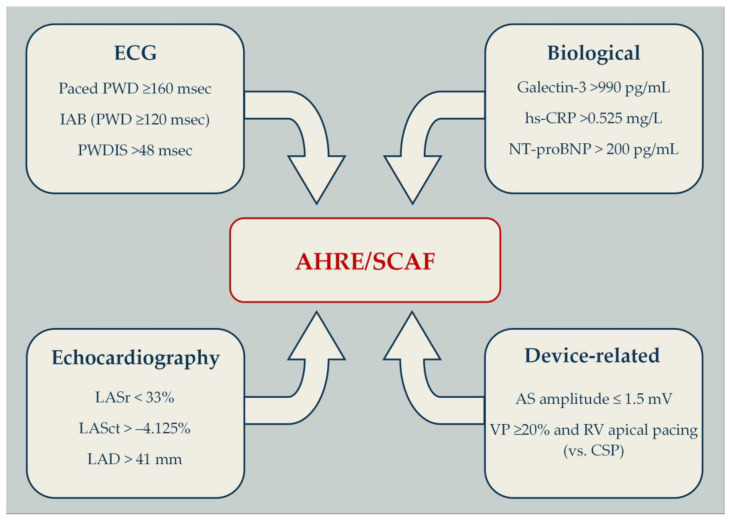
Threshold-based predictors of device-detected AHRE/SCAF. AS—atrial sensing; AHRE—atrial high-rate episode; CSP—conduction system pacing; IAB—interatrial block; hs-CRP—high-sensitivity C-reactive protein; LAD—left atrial diameter; LASr—Left atrial reservoir strain; LASct—left atrial conduit strain; NT-proBNP—N-terminal pro-B-type natriuretic peptide; PWD—P-wave duration; PWDIS—P-wave dispersion; RV—right ventricle; SCAF—subclinical atrial fibrillation; VP—ventricular pacing.

Detailed results from the studies included in this review are provided in Supplementary Table S1. Among the proposed predictors, only a subset has demonstrated reproducible, clinically meaningful cut-off values across studies. These threshold-based parameters, which may support translation into actionable clinical decision-making, are summarized in Figure 2.

In light of these findings, several predictors demonstrate broadly consistent and complementary associations across different study designs. Among clinical variables, increasing age remains the most reproducible determinant of AHRE/SCAF occurrence and of progression toward longer episodes and/or overt clinical atrial fibrillation [12,13]. Similarly, excess body weight is repeatedly associated with AHRE occurrence and progression to higher-burden phenotypes [12,14,15]. HF-related indices also demonstrate a concordant bidirectional relationship, with HF predicting AHRE/SCAF and AHRE burden predicting subsequent HF worsening or hospitalization in CIED cohorts, including CRT-D recipients [17,19,20,21]. In parallel, refinements of atrial electrical substrate quantification (e.g., interatrial block and advanced P-wave indices, including paced metrics) show largely consistent associations with AHRE, and dedicated indices with proposed cut-offs have also been reported [5,32,33,35,36]. Imaging findings similarly converge on atrial remodelling; beyond “size-only” assessment, LA functional impairment (LA strain) has emerged as a reproducible marker, while LA size remains a broadly consistent structural anchor across cohorts [39,40,41,42,43]. Finally, emerging device-derived signals (e.g., atrial sensing amplitude trends) and pacing-related exposure/strategy appear concordant in multiple reports, supporting the role of device analytics and pacing-related atrial–ventricular interactions in AHRE susceptibility [71,72,73,74,75,76].

Conversely, several associations remain contradictory or context-dependent. Hypertension and diabetes are frequent in CIED populations, yet their independent association with incident AHRE is inconsistent and often attenuates after multivariable adjustment, suggesting confounding and/or mediation by atrial remodelling and comorbidity clustering [16,17,18,22,23,24]. LVEF shows non-uniform associations across cohorts, indicating limited sensitivity of LVEF as a standalone predictor [3,60]. Biomarker signals also vary; profibrotic and inflammatory markers (including Galectin-3 and hs-CRP) show promising but not uniformly replicated associations across populations and adjustment strategies [62,63,64]. Furthermore, NT-proBNP and high-sensitivity troponin show context-dependent associations; they may differentiate patients with recent short AHRE in selected pacemaker cohorts [67], yet they appear most informative for higher-burden phenotypes (e.g., episodes ≥ 24 h) in ILR screening [68], while other prospective pacemaker analyses report no independent predictive value [64].

## 4. Future Perspectives

Rapid advancements in digital health technologies are reshaping the landscape of subclinical AF detection. Wearable devices and home-based monitoring platforms now enable continuous or intermittent rhythm assessment outside the clinical setting, facilitating earlier recognition of arrhythmic episodes. The incorporation of artificial intelligence (AI) and machine learning algorithms into signal interpretation holds promise for improving diagnostic accuracy, predicting arrhythmic risk, and personalizing follow-up strategies. Future research should focus on validating these technologies across diverse populations and determining how device-generated data can be integrated into clinical decision pathways, particularly regarding anticoagulation and rhythm-control interventions.

## 5. Conclusions

Device-detected subclinical atrial fibrillation is increasingly recognized as a clinically relevant condition, with important implications for stroke prevention, HF risk, and early rhythm management. A wide spectrum of predictors—encompassing clinical characteristics, electrocardiographic findings, echocardiographic parameters, and circulating biomarkers—supports a more refined approach to surveillance and risk stratification. Integrating these complementary markers may enhance the identification of individuals most likely to benefit from intensified monitoring or early therapeutic intervention. Continued research is needed to establish clear risk thresholds, optimize management algorithms, and evaluate long-term outcomes associated with proactive treatment of SCAF.

## Figures and Tables

**Table 1:** Key predictors of device-detected AHRE/SCAF in CIED patients.

Domain	Predictor	Association with SCAF/AHRE	Notes
**Clinical**	Older age	Higher risk of first AHRE; progression from short SCAF to SCAF > 24 h or clinical AF [12,13,17,19]	Most consistent clinical predictor across CIED/ILR cohorts [12,13,17,19]
	Higher BMI	More AHRE; progression from SCAF > 6 min to SCAF > 24 h or clinical AF [12,14]	Correlated with LA enlargement, epicardial fat, inflammation, and neurohormonal activation [14,15]
	HF	Higher SCAF prevalence; independent predictor of AHRE > 24 h; AHRE linked to HF worsening or HF hospitalization [17,19,20,21]	Bidirectional HF–SCAF relationship in CIED and CRT-D populations [17,19,20,21]
	OSA	2–3-fold higher risk of SCAF ≥ 6 h/day; longer daily AHRE burden with increasing OSA severity [25,26,27]	Dynamic trigger; associated with hypoxia, pressure swings, sympathetic activation, fibrosis [25,26,27,28]
**ECG**	PWD, IAB, paced PWD	Prolonged PWD/IAB and paced PWD > 160 ms associated with SCAF/AHRE; MVP score predicts long AHRE (>24 h) [5,32,33,35]	Markers of atrial electrical and structural remodelling [5,32,33,35]
	PWDIS	Higher PWDIS independently predicts new-onset AHRE; cut-off ≈ 48 ms [36]	Reflects heterogeneous atrial conduction [36]
**Echocardiography**	LA strain	Lower LASr/LASct associated with SCAF/AHRE; LASr < 33% identifies higher risk even without LA enlargement [41,42]	Sensitive marker of atrial cardiomyopathy; correlates with CMR fibrosis [39,40,41,42,43]
	LA size	Larger LAD/LAVI associated with AHRE; LAD > 41 mm predicts AHRE > 6 min and >6 h [16,50,51,52]	Robust routine echo parameter for AHRE prediction [16,51,52]
	GLS	Less negative GLS independently predicts AHRE after dual-chamber pacemaker implantation [61]	Captures subtle LV systolic dysfunction beyond LVEF [3,60,61]
**Biomarkers**	Galectin-3	Higher levels associated with incident AHRE and greater AHRE burden [62,63]	Pro-fibrotic marker; independent in CRT cohorts, but inconsistent in pacemakers [62,63]
	hs-CRP	hs-CRP > 0.525 mg/L predicts AHRE ≥ 6 min and higher AHRE burden; CRP independently predicts AHRE onset [13,64]	Integrates chronic inflammatory remodelling [13,64]
	NT-proBNP	Higher levels associated with recent AHRE ≥ 6 min/paroxysmal AF in pacemakers and with SCAF ≥ 24 h in ILR cohorts [67,68]	Marker of myocardial stretch; strongest for longer AF burden [67,68]
**Device & pacing**	Low atrial sensing amplitude	Atrial sensing amplitudes ≤ 1.5 mV predict AHRE > 24 h, and amplitudes < 2.45 mV in the first month after implant predict progression of AHRE duration [71,72]	Atrial sensing amplitude often declines gradually in the year before the first AHRE [71,72]
	High ventricular pacing burden and pacing site	Ventricular pacing ≥ 20% and RV apical pacing associated with more AHRE and lower LVEF as compared to CSP/LBBAP [73,74,75,76]	Minimizing ventricular dyssynchrony via conduction-system pacing reduces AHRE risk [73,74,75,76]

Key predictors of device-detected AHRE and SCAF in continuously monitored patients. Predictors are grouped into clinical, ECG, echocardiographic, biomarker-based, and device/pacing domains. AF—atrial fibrillation; AHRE—atrial high-rate episode; BMI—body mass index; CIED—cardiac implantable electronic device; CMR—cardiac magnetic resonance; CRT—cardiac resynchronization therapy; CRT-D—cardiac resynchronization therapy defibrillator; CSP—conduction system pacing; ECG—electrocardiogram; GLS—global longitudinal strain; HF—heart failure; hs-CRP—high-sensitivity C-reactive protein; IAB—interatrial block; ILR—implantable loop recorder; LA—left atrial; LAD—left atrial diameter; LASr—left atrial reservoir strain; LASct—left atrial conduit strain; LAVI—left atrial volume index; LBBAP—left bundle branch area pacing; LV—left ventricular; LVEF—left ventricular ejection fraction; NT-pro-BNP—N-terminal pro-B-type natriuretic peptide; OSA—obstructive sleep apnea; PWD—P-wave duration; PWDIS—P-wave dispersion; SCAF—subclinical atrial fibrillation.

## Data Availability

Study data are available, upon reasonable request, from the corresponding author (larisa.anghel@umfiasi.ro). A justification for its further use should be provided.

## Supplementary Materials

The following supporting information can be downloaded at https://www.mdpi.com/article/10.3390/jcm15020578/s1: Table S1: Summary of included studies related to AHRE/SCAF.

## Abbreviations

The following abbreviations are used in this manuscript:

AF	Atrial fibrillation
AHRE	Atrial high-rate episode
AI	Artificial intelligence
BMI	Body mass index
CIED	Cardiac implantable electronic device
CIEDs	Cardiac implantable electronic devices
CMR	Cardiac magnetic resonance
CRP	C-reactive protein
CRT	Cardiac resynchronization therapy
CRT-D	Cardiac resynchronization therapy defibrillator
CSP	Conduction system pacing
CT	Computed tomography
DWS	Diastolic wall strain
ECG	Electrocardiogram
GLS	Global longitudinal strain
HF	Heart failure
HR	Hazard ratio
hs-CRP	High-sensitivity C-reactive protein
ICD	Implantable cardiac defibrillator
IAB	Interatrial block
ILR	Implantable loop recorder
ILRs	Implantable loop recorders
LA	Left atrium/left atrial
LAA	Left atrial appendage
LACI	Left atrial coupling index
LAD	Left atrial diameter
LASr	Left atrial reservoir strain
LASct	Left atrial conduit strain
LAVI	Left atrial volume index
LGE	Late gadolinium enhancement
LBBAP	Left bundle branch area pacing
LV	Left ventricle/left ventricular
LVEF	Left ventricular ejection fraction
MHR	Monocyte-to-high-density lipoprotein ratio
MVP score	Morphology–voltage–P-wave score
NT-proBNP	N-terminal pro–B-type natriuretic peptide
OSA	Obstructive sleep apnoea
OR	Odds ratio
PWA	P-wave axis
PWD	P-wave duration
PWDIS	P-wave dispersion
PWIs	P-wave indices
PWPT	P-wave peak time
PWPTV1	P-wave peak time in lead V1
PWPTD2	P-wave peak time in lead II
PTF	P-wave terminal force in lead V1
PWV	P-wave voltage in lead I
QRS	QRS complex
QTc	Corrected QT interval
ROC	Receiver operating characteristic
RVP	Right ventricular pacing
SCAF	Subclinical atrial fibrillation
TIA	Transient ischaemic attack
MeSH	Medical subject headings
QRS-T angle	Angle between QRS and T-wave vectors on ECG
