# Supplementary material for: Predictors for Device-Detected Subclinical Atrial Fibrillation: An Up-to-Date Narrative Review
Source: J Clin Med. 2026 Jan 11;15(2):578. doi: 10.3390/jcm15020578 (PMC12841777; doi:10.3390/jcm15020578)
Supplement: Supplementary file 1 [file jcm-15-00578-s001.zip › jcm-4071930-supplementary.pdf]

Review

Predictors for Device-Detected Subclinical Atrial Fibrillation: An Up-to-Date Narrative Review – supplemental data

Traian Chiuariu [1,2], Larisa Anghel [1,2,*], Delia Melania Popa [1,2], Gavril-Silviu Bîrgoan [1,2], Șerban Daniel Fechet [1,2], Răzvan-Liviu Zanfirescu [2,3], Mircea Ovanez Balasanian [1,2], Radu Andy Sascău [1,2] and Cristian Stătescu [1,2]

Supplemental Table S1. Summary of included studies related to AHRE/SCAF

Study (first author, year)	Study title	Study design/type	Key findings highlighted in this review
Park YJ, 2021 [2]	Subclinical Atrial Fibrillation Burden and Adverse Clinical Outcomes in Patients With Permanent Pacemakers	Retrospective observational study	In 496 permanent pacemaker patients without pre-existing AF (median follow-up 5.2 years), a high 6-month SCAF burden (≥24 h in at least one 6-month interval) was associated with the highest risk of composite adverse outcomes and was mainly driven by progression to clinical AF and ischemic stroke; high-burden SCAF independently predicted composite adverse outcomes (OR 20.1) and progression to clinical AF (OR 36.2), and SCAF episodes preceded ischemic stroke events.
Lu W-D, 2021 [3]	Atrial high-rate episodes and risk of major adverse cardiovascular events in patients with dual chamber permanent pacemakers: a retrospective study	Retrospective observational study	In 481 dual-chamber permanent pacemaker recipients (median follow-up ~40 months), device-detected AHRE ≥5 min and ≥6 h were independently associated with MACE (HR 5.252 and HR 2.548, respectively), whereas AHRE ≥24 h was not significant in the overall cohort; the duration threshold associated with MACE varied by clinical history, with AHRE ≥5 min remaining

			the key signal in patients with prior AF and in those with prior myocardial infarction.
Kaufman ES, 2012 [4]	Positive predictive value of device-detected atrial high-rate episodes at different rates and durations: An analysis from ASSERT	Secondary/post hoc analysis of an RCT cohort	CIED-detected AHRE are associated with an increased risk of stroke, but the risk is significantly lower than the stroke risk of clinical AF. CIEDs have the ability to monitor, store and interpret complex arrhythmias, which has generated the new arrhythmic entity: AHRE.
Healey Jeff S, 2012 [5]	Subclinical Atrial Fibrillation and the Risk of Stroke (ASSERT)	RCT	Although several ECG variables were associated with AF occurrence, only prolonged PWD and the presence of IAB independently predicted episodes lasting >24 hours – durations known to confer increased thromboembolic risk. Despite widespread use of the term, there is no uniform definition of AHRE.
Miyazawa K, 2022 [6]	Characteristics of patients with atrial high rate episodes detected by implanted defibrillator and resynchronization devices	Post hoc analysis of the IMPACT trial cohort	Despite widespread use of the term, there is no uniform definition of AHRE; different studies and device manufacturers apply atrial rate thresholds ranging from >170 beats/min, >175 beats/min, >190 beats/min to >200 beats/min.
Nakano M, 2019 [7]	Impact of atrial high-rate episodes on the risk of future stroke	Retrospective cohort study	Despite widespread use of the term, there is no uniform definition of AHRE; Likewise, the minimum episode duration varies, with commonly used cut-offs of 30 seconds, 5 minutes or 6 minutes,.
Wilton SB, 2016 [8]	Frequency and Outcomes of Postrandomization Atrial Tachyarrhythmias in the Resynchronization/Defibrillation in Ambulatory Heart Failure Trial	Secondary/post hoc analysis of an RCT cohort	Likewise, the minimum episode duration varies, with commonly used cut-offs of 30 seconds, 5 minutes or 6 minutes, most of which derive from investigations linking AHRE duration to thromboembolic risk.
Li Y-G, 2019 [9]	Atrial high-rate episodes and thromboembolism in patients without atrial fibrillation: The West Birmingham Atrial Fibrillation Project	Retrospective observational study	Likewise, the minimum episode duration varies, with commonly used cut-offs of 30 seconds, 5 minutes or 6 minutes, most of which derive from investigations linking AHRE duration to thromboembolic risk.
Wong JA, 2018 [12]	Progression of Device-Detected Subclinical Atrial Fibrillation and the Risk of Heart Failure	Post hoc analysis of the ASSERT trial cohort	Beyond onset, age also tracks disease trajectory: in a prospective device-monitoring program, older age independently predicted progression from brief subclinical AF to sustained episodes (>24 h) or clinical AF, a transition that carries higher thromboembolic relevance.
Pastori D, 2018 [13]	Inflammation and the risk of atrial high-rate episodes (AHREs) in patients with cardiac implantable electronic devices	Retrospective observational study	AHREs are associated with an increased risk of developing AF and thromboembolism. Multivariable Cox regression analysis showed that age [HR 1.020, 95% CI 1.004–1.035, p = 0.011] and prior AF (HR 3.521, 95% CI 2.831–5.206, p < 0.001) are associated with AHRE occurrence.

Çinier G, 2018 [14]	Increased Body Mass Index is Associated with Device Detected Silent Atrial Fibrillation	Prospective observational study	In 449 dual-chamber pacemaker recipients, AHREs (atrial rate >220 bpm lasting >5 minutes) were detected in 28.5%. On multivariable analysis, AHRE occurrence was independently associated with higher BMI, older age, higher mean resting heart rate, larger left-atrial anteroposterior diameter, and a higher CHA_2DS_2-VASc score.
Kim M, 2021 [16]	Prevalence and Predictors of Clinically Relevant Atrial High-Rate Episodes in Patients with Cardiac Implantable Electronic Devices	Prospective multicenter observational study	In a prospective multicenter cohort of 816 CIED patients without prior AF, AHREs were defined as a programmed atrial detection rate >220 beats/min. During a median 18-month follow-up, the longest-duration AHRE exceeded >15 seconds, >6 minutes, and >6 hours in 30.1%, 13.7%, and 9.4% of patients, while clinically documented AF occurred in 6.0%. For AHREs >6 minutes, LA diameter >41 mm (OR 2.08; 95% CI 1.25–3.45) and pre-implant sick sinus syndrome (OR 3.22; 95% CI 1.91–5.43) were independently associated.
Li YG, 2021 [17]	Identifying At-Risk Patients for Sustained Atrial High-Rate Episodes Using the C_2HEST Score: The West Birmingham Atrial Fibrillation Project	Retrospective observational study	In 500 consecutive CIED patients without AF, sustained AHRE (>24 h) occurred in 44 (8.8%) after a mean follow-up of 3.1 months. Risk increased with the C_2HEST score (≈39% higher risk per point), and C2HEST ≥4 identified a high-risk subgroup (incidence 36.2% per year; 95% CI 2.14–5.16); discrimination was moderate (AUC 0.73; 95% CI 0.64–0.81) and superior to CHA2DS2-VASc/CHADS2/HATCH.
Chen JH, 2021 [18]	Atrial High-Rate Event Incidence and Predictors in Patients With Permanent Pacemaker Implantation	Retrospective observational study	In 289 dual-chamber pacemaker recipients without prior AF, AHREs (≥175 bpm, duration ≥5 min) were detected in 31.5% over 12 months. Independent predictors were older age (OR 1.041; 95% CI 1.018–1.064; p<0.001), implantation for symptomatic sick sinus syndrome (OR 2.225; 95% CI 1.227–4.036; p=0.008), and a higher percentage of atrial pacing (OR 1.010; 95% CI 1.002–1.017; p=0.016).
Arai S, 2020 [20]	Relationship between device-detected subclinical atrial fibrillation and heart failure in patients with cardiac resynchronization therapy defibrillator	Retrospective observational study	In 153 CRT-D recipients with LVEF <35% followed for 50 months, HF hospitalization was more frequent in those with subclinical AF than in those with clinical AF or no AF (70% vs 49% vs 38%; log-rank p=0.03). Subclinical AF remained a predictor of HF hospitalization on multivariable analysis and coincided with lower biventricular pacing percentages and more inappropriate therapy.
Nishinarita R, 2019 [21]	Burden of Implanted-Device-Detected Atrial High-Rate Episode Is Associated With Future Heart Failure Events - Clinical Significance of Asymptomatic	Retrospective observational study	In 104 CIED recipients without prior AF, 33% exhibited AHRE during the first year after implantation. New-onset AHRE and a higher AHRE burden were independent predictors of subsequent worsening HF, with excess risk concentrated in the high-burden group (overall worsening HF 12%).

	Atrial Fibrillation in Patients With Implantable Cardiac Electronic Devices		
Valdez Baez FJ, 2025 [22]	Clinical- and Device-Related Factors Associated With Atrial High Rate Episodes in Patients With Dual-Chamber Pacemakers	Retrospective observational study	In 450 dual-chamber pacemaker patients, AHREs were present in 41.1%. Multivariable analysis associated AHREs with female sex (OR 1.63; p=0.020), prior AF/AT (OR 2.95; p<0.001), follow-up >90 days (OR 4.14; p=0.012), and use of AAI-DDD pacing mode (OR 1.92; p=0.027).
Çakmak Karaaslan Ö, 2025 [23]	Relationship between predictive factors and atrial high-rate episodes in heart failure with reduced ejection fraction patients with cardiac implantable electronic devices	Retrospective observational study	In 155 HFrEF patients with CIEDs and no prior AF, AHRE presence was linked to lower LVEF and larger left-atrial diameter, along with higher creatinine, uric acid, and TSH levels. In multivariable logistic regression, lower LVEF (HR 0.890; 95% CI 0.795–0.998; p=0.046), larger LA diameter (HR 8.215; 95% CI 1.557–43.34; p<0.001), and higher uric acid (HR 1.650; 95% CI 1.063–2.561; p=0.025) independently predicted AHRE.
Malanchini G, 2025 [24]	Association between device-detected subclinical atrial fibrillation and diabetes in patients with implantable cardioverter-defibrillators: A propensity score-matched analysis	Retrospective propensity score-matched analysis	Among 1,619 ICD/CRT-D recipients without prior AF (median follow-up 2.3 years; 963 [22.4%] with diabetes), diabetes was associated with a higher incidence of DDAF ≥15 min (3-year 65.7% vs 57.7%; p=0.032) and ≥5 h (62.0% vs 52.2%; p=0.010), whereas no differences were observed for ≥24 h or ≥7 days in unmatched or matched analyses. Using a landmark approach, DDAF was associated with increased mortality in diabetic patients (HR 4.20; 95% CI 1.89–9.33; p<0.001) but not in non-diabetics (HR 1.76; 95% CI 0.89–3.46; p=0.099).
Moubarak G, 2017 [25]	Variability in obstructive sleep apnea: Analysis of pacemaker-detected respiratory disturbances	Prospective observational study	In 58 dual-chamber pacemaker patients with a pacemaker-derived RDI, valid nightly RDI measurements were available in 98% of nights and showed substantial night-to-night variability. Neither single-night RDI values nor fixed cutoffs predicted AF occurrence, supporting a burden-based interpretation of sleep-disordered breathing.
La Greca C, 2025 [26]	Dynamic evaluation of atrial fibrillation risk factors using multiple sensors of implantable cardioverter defibrillators	Prospective observational study	In 411 HF patients with ICDs followed for a median of 26 months, AHRE burden ≥5 min/day occurred in 34% and ≥23 h/day in 17%. HeartLogic "IN-alert" HF status and reduced physical activity (≥1 h/day below usual) were independently associated with both AHRE thresholds, while severe sleep apnoea (RDI ≥30 events/hour) was associated with AHRE ≥5 min/day only. A composite sensor-based score (2×IN-alert + 1×RDI≥30 + 4×activity reduction for ≥5 min/day; 1×IN-alert + 2×activity reduction for ≥23 h/day) stratified AHRE risk, with higher strata showing markedly higher incidence rate ratios (6.75 and 11.46, respectively).

Rajaratnam D, 2025 [27]	Association Between Device-Detected Obstructive Sleep Apnea and Atrial Arrhythmia Burden in Patients Monitored by HeartLogic-Enabled Cardiac Devices: A Retrospective Cohort Study	Retrospective cohort study	In a HeartLogic-monitored retrospective ICD/CRT-D cohort, increasing device-estimated OSA severity (AP Scan-derived AHI equivalent) correlated with longer daily atrial arrhythmia/AHRE duration. Each 1-unit increase in AHI was associated with ~10.55 additional seconds/day of atrial arrhythmia burden (p=0.005). Compared with no sleep apnoea, mild/moderate/severe AHI categories showed substantially longer daily arrhythmia durations (+2.8, +2.8, and +3.0 hours/day; all p<0.001).
Chen JY, 2022 [29]	The performance of five models compared with atrial high rate episodes predicts new atrial fibrillation after cardiac implantable electronic devices implantation	Retrospective observational study	Among 470 CIED recipients without prior AF (median follow-up 29 months), 34 developed new AF (incidence 2.99/100 patient-years; 95% CI 1.67–6.20). In multivariable Cox models, AHRE ≥6 min, AHRE ≥24 h, and the HAT_2CH_2 score independently predicted incident AF. An AHRE duration cut-off of 9.3 minutes yielded good discrimination (AUC 0.806; 95% CI 0.722–0.889; p<0.001), and AF incidence was ~7-fold higher above this threshold.
Tekkeşin A, 2017 [31]	Diastolic Electrocardiographic Parameters Predict Implantable Device Detected Asymptomatic Atrial Fibrillation	Cross-sectional study	In 203 dual-chamber device patients, 51 (25.1%) had AHREs. PQ and corrected QT intervals were longer in those with AHREs, while Tend-P and Tend-Q were longer in those without AHREs. In multivariable analysis, the diastolic electrocardiography index, heart rate, and PQ/QT intervals were independent predictors of AHREs, supporting "diastolic ECG" markers as a practical risk signal for device-detected asymptomatic AF/AHRE.
Xing LY, 2023 [32]	Electrocardiographic markers of subclinical atrial fibrillation detected by implantable loop recorder: insights from the LOOP Study	Secondary/post hoc analysis of an RCT cohort	In the LOOP Study ILR-screened cohort (n=1370), 419 (30.6%) developed device-detected AF, with a mean cumulative AF burden of ~1.5%. Baseline ECG substrate markers were linked to AF onset and burden, with P-wave duration (non-linear; both short and long PWD), P-wave terminal force in V1, and interatrial block showing stronger associations with long-lasting AF (≥24 h). Among participants with AF, cumulative burden tended to decline over time (IRR 0.70; 95% CI 0.51–0.96), while interatrial block predicted progression to AF ≥24 h (HR 1.86; 95% CI 1.02–3.39); longer PWD also tracked with progression on spline analyses.
Campal JMR, 2022 [33]	Paced P-wave duration as a significant predictor for atrial high-rate episodes in patients with cardiac implantable electronic devices	Retrospective observational study	In 220 dual-chamber CIED patients without prior documented AF (mean follow-up 59 months), atrial pacing substantially prolonged P-wave duration versus sinus rhythm (154±27 vs 115±18 ms; p<0.001). Both sinus and paced P-waves were longer in patients who later developed AHREs, and a paced P-wave ≥160 ms provided the strongest prediction—particularly for episodes

			>24 h (OR 4.2; 95% CI 1.6–11.4; p=0.004). The authors propose paced PWD ≥160 ms as a practical IAB definition in paced rhythms.
Karakayali M, 2023 [34]	Assessment of the efficacy of the electrocardiographic P-wave peak time in predicting atrial high rate episode in patients with cardiac implantable electronic devices	Cross-sectional study	In 158 CIED patients stratified by AHRE presence on interrogation, conventional P-wave indices (PWDmin, PWDmax, and PWDis) did not differ significantly between groups. In contrast, P-wave peak time in V1 (PWPTV1) and in lead II (PWPTD2) were significantly higher in the AHRE group. These "P-wave peak time" metrics were highlighted as novel ECG markers with potential prognostic value for future AHRE development.
Zhu W, 2025 [35]	Association of P-wave characteristics with long-standing atrial high rate episodes (AHREs) following pacemaker implantation	Retrospective observational study	In 180 pacemaker recipients, the AHRE group showed higher atrial and ventricular pacing burdens, with longer sinus/paced PWPT, longer sinus PWDis, and higher paced MVP scores. High ventricular pacing percentage was an independent risk factor for AHRE (p=0.019). For long-standing AHRE (≥24 h), sick sinus syndrome and advanced interatrial block were more frequent, and high paced MVP score and prolonged paced PWPT independently predicted ≥24 h episodes (p=0.006 and p=0.007).
Nishinarita R, 2021 [36]	Novel Predictor for New-Onset Atrial High-Rate Episode in Patients With a Dual-Chamber Pacemaker	Retrospective observational study	In 101 CIED patients without prior AF followed for 1 year after implantation, PWDis in baseline sinus ECGs was markedly higher in those who developed AHREs (62.6±13.1 vs 38.2±13.9 ms; p<0.0001). PWDis remained an independent predictor in logistic regression (OR 1.11; 95% CI 1.06–1.17; p<0.0001). ROC analysis suggested a PWDis cut-off of 48 ms (sensitivity 73.8%, specificity 77.9%) for identifying patients at higher AHRE risk.
Habibi M, 2015 [39]	Association of left atrial function and left atrial enhancement in patients with atrial fibrillation: cardiac magnetic resonance study	Prospective observational study	In 90 AF patients referred for ablation and 14 healthy volunteers, cine CMR feature-tracking showed that persistent AF was associated with larger LA size (LAVImax 56±17 vs 49±13 mL/m^2, p=0.036) and higher LA LGE burden (~36.8±14.8% vs ~27.1±11.7%, p<0.001) than paroxysmal AF. LA functional indices (including passive emptying fraction and strain/strain-rate measures such as PLAS, SR-s, SR-ed, SR-ld) were lower in persistent AF and higher in healthy controls; higher LGE burden remained associated with worse LA reservoir/conduit/booster-pump function in multivariable analyses (p<0.05).
Peters DC, 2017 [40]	CMR-Verified Lower LA Strain in the Presence of Regional Atrial Fibrosis in Atrial Fibrillation	Prospective observational study	In 18 AF patients and 12 controls undergoing 3D LGE CMR and cine CMR, regional LA strain was lower in LGE-enhanced (fibrosis-suggestive) atrial regions than in non-enhanced regions (14±11% vs 20±12%, p=0.007). A strain threshold <16% showed only modest discrimination for LGE-positive

			regions (AUC 0.66, p=0.11), while global strain correlated strongly with LA size/function and only modestly with overall LGE burden (r≈−0.36, p=0.049).
Olsen FJ, 2024 [41]	Left Atrial Strain Predicts Subclinical Atrial Fibrillation Detected by Long-term Continuous Monitoring in Elderly High-Risk Individuals	Prospective observational study	In an elderly high-risk cohort with long-term continuous rhythm monitoring, lower LA reservoir strain remained independently associated with incident subclinical AF after multivariable adjustment (HR 1.04 [1.02–1.05] per 1% decrease), and contraction strain showed a similar independent association. Lower reservoir strain was accompanied by higher subclinical AF incidence rates (e.g., 14.5 vs 9.8 events/100 person-years for low vs high reservoir strain).
Wang P, 2024 [42]	Study on the relationship between atrial high-rate episode and left atrial strain in patients with cardiac implantable electronic device	Cross-sectional study	In 105 CIED patients (AHRE+ n=40; AHRE− n=65), LA strain analysis showed that LASct was the only LA strain parameter independently associated with AHRE after adjustment for clinical covariates (OR 1.18, 95% CI 1.01–1.38; p=0.041). ROC analysis suggested a LASct cut-off of −4.125% for predicting AHRE (sensitivity 37.5%, specificity 87.7%).
Huang SH, 2023 [43]	Multimodality imaging assessment of the Biatrial remodeling of the burden of atrial high-rate episodes in patients with cardiac implanted electronic devices	Retrospective observational study	In CIED patients stratified by AHRE duration (<6 min, 6 min–6 h, >6 h), longer AHRE duration tracked with progressively worse atrial remodeling (trend p<0.05), including larger LA volumes and impaired LA function/strain (PALS/PACS). Compared with AHRE <6 min, AHRE >6 h was associated with higher indexed LA maximal and minimal volumes (LAVmax 57.1 vs 45.4 mL/m², p=0.002; LAVmin 42.6 vs 28.2 mL/m², p<0.001) and lower total LAEF (28.0% vs 38.2%, p=0.004); conclusions highlighted that functional remodeling appears after AHRE >6 min, with more pronounced structural/functional deterioration when AHRE exceed 6 h.
Saberniak J, 2023 [44]	Left atrial appendage strain predicts subclinical atrial fibrillation in embolic strokes of undetermined source	Prospective observational study	In 185 patients with embolic stroke of undetermined source (ESUS) without known AF, ILR follow-up identified subclinical AF in 60 (32%). LAA strain was markedly lower in those who developed subclinical AF (LAA reservoir strain 19.2±4.5% vs 25.6±6.5%, p<0.001), with higher LAA mechanical dispersion (34±24 ms vs 26±20 ms, p=0.02), while phasic LA strain did not differ significantly. LAA reservoir strain showed strong discrimination for subclinical AF (AUC 0.80, 95% CI 0.73–0.87; sensitivity 80%, specificity 73%), and both LAA reservoir strain and LAA mechanical dispersion provided independent/incremental prediction.

Chen L, 2024 [45]	Automatic 3D left atrial strain extraction framework on cardiac computed tomography	Retrospective observational study	In 111 patients undergoing ECG-gated contrast-enhanced cardiac CT for evaluation of subclinical AF, a fully automated deep-learning pipeline enabled 3D LA segmentation and CT-based LA strain extraction (segmentation DSC 0.9603). CT-derived LA volumes and emptying fraction showed excellent agreement with expert measurements (ICC 0.949 for minimal LA volume; 0.904 for maximal LA volume; 0.902 for LAEF), and CT-derived LA strain had moderate agreement with 2D echo strain (ICCs >0.703). Using AHRE burden as an AF-burden surrogate, AHRE >6 minutes was associated with significantly lower CT-derived global LA strain and LAEF versus AHRE ≤6 minutes.
Wattanachayakul P, 2025 [50]	Left Atrial Size and Left Atrial Volume Index as Predictors of Atrial High-Rate Episodes	Meta-analysis	In a meta-analysis of 18 cohort studies in CIED patients without prior AF at implantation, those with AHRE had larger left atria on transthoracic echocardiography than those without AHRE. Pooled mean differences were +2.19 mm for LA diameter (95% CI 1.11–3.28; I^2=80%; p<0.001) and +4.88 mL/m² for LAVI (95% CI 2.55–7.21; I^2=55%; p<0.001), supporting LA size/LAVI as consistent echocardiographic correlates of AHRE occurrence.
Chen J-Y, 2021 [51]	Atrial high-rate episodes predict major adverse cardio/cerebrovascular events in patients with cardiac implantable electrical devices	Retrospective observational study	In 470 consecutive CIED recipients (median age 76 years; median follow-up 29 months), AHRE ≥6 min, ≥6 h, and ≥24 h occurred in 26.8%, 13.4%, and 8.3%, respectively, and MACCE occurred in 123 patients (142 events). A 6-minute threshold provided the best MACCE discrimination (highest Youden index), and AHRE ≥6 min–24 h was linked to MACCE and predicted subsequent clinical AF. In multivariable Cox models adjusting for CHA2DS2-VASc, AHRE ≥6 min (HR 2.254; 95% CI 1.574–3.226; p<0.001), AHRE ≥6 h (HR 2.515; 95% CI 1.688–3.746; p<0.001), and AHRE ≥24 h (HR 2.185; 95% CI 1.364–3.501; p=0.001) remained independently associated with MACCE; male sex, lower BMI, and larger LA diameter were independently associated with AHRE development.
Arslan S BKB, 2022 [52]	The Relationship between Intracardiac Atrial Sensing Values and Atrial High-rate Episodes in Patients With Permanent Pacemakers Implanted due to Sick Sinus Syndrome	Retrospective observational study	In a retrospective dual-chamber (DDDR) pacemaker cohort implanted for sick sinus syndrome (2011–2019), at least one AHRE was detected in 51.7%. The AHRE group had larger LA diameter and lower atrial (P-wave) sensing amplitudes. On multivariable time-to-event analysis, LA diameter (HR 1.10; 95% CI 1.04–1.18; p<0.01) and P-wave sensing amplitude (HR 0.74; 95% CI 0.57–0.95; p=0.02) independently predicted AHRE, indicating that lower baseline atrial sensing and LA enlargement track with higher AHRE risk.

Arslan A, 2025 [57]	Relationship Between Left Atrial Coupling Index and Atrial High-Rate Episodes	Retrospective observational study	In 203 CIED patients with echocardiography available ≥6 months before assessment, AHRE occurred in 42.8% (n=87). Higher mitral E/Em, LAVI, LACI, and LVEDD and lower LVEF characterized the AHRE group; on multivariable analysis, LACI independently predicted AHRE (OR 1.752; 95% CI 1.356–2.263; p<0.001) alongside LAVI and age, and ROC comparison indicated superior discrimination for LACI versus LAVI.
Bufano G, 2022 [58]	Predictive Value of Left Atrial and Ventricular Strain for the Detection of Atrial Fibrillation in Patients With Cryptogenic Stroke	Prospective observational study	In 72 patients with cryptogenic stroke undergoing ILR surveillance, subclinical AF was detected in 23 (32%) at a mean of 6.5 months. Multivariable regression identified peak atrial contraction strain (adjusted OR 0.72; 95% CI 0.48–0.90; p=0.005) and LV strain (adjusted OR 0.69; 95% CI 0.46–0.95; p=0.041) as independent correlates of AF occurrence.
Kishima H, 2021 [59]	Left ventricular stiffness assessed by diastolic Wall strain predicts asymptomatic atrial high-rate episodes in patients with pacemaker implantation	Retrospective observational study	In 147 pacemaker-implanted patients, AHRE (atrial rate >180 bpm, duration >6 min) were detected in 50/147 during 38.3±13.8 months of follow-up. The AHRE group had lower diastolic wall strain (DWS 0.29±0.07 vs 0.39±0.06; p<0.0001), and multivariable analysis retained DWS as the only independent factor associated with AHRE; DWS <0.33 marked a higher AHRE incidence.
Samuel J [60]	Incidence of atrial high rate episodes after dual-chamber permanent pacemaker implantation and its clinical predictors	Cross-sectional study	In 100 dual-chamber PPI recipients without prior AF, device interrogation (starting ≥1 month post-implant) identified AHRE in 17% over a mean follow-up of 15.2±7.5 months. Right ventricular apical lead position showed an independent association with AHRE (OR 3.50; 95% CI 1.02–12.03; p=0.04).
Le Nguyen SK, 2024 [61]	The incidence and risk factors of atrial high-rate episodes in patients with a dual-chamber pacemaker	Prospective observational study	In 145 dual-chamber pacemaker recipients followed for 6 months, AHRE occurred in 30.3%. Independent predictors in multivariable Cox models included pre-implant antiarrhythmic drug use (HR 7.71; 95% CI 2.58–23.02; p<0.001), history of paroxysmal SVT (HR 2.45; 95% CI 1.18–5.09; p=0.016), higher PAC percentage on 24-h Holter (HR 1.008; 95% CI 1.003–1.014; p=0.003), and LV GLS (HR 0.92; 95% CI 0.84–0.99; p=0.049).
Aksan G, 2021 [62]	Galectin-3 levels and the prediction of atrial high-rate episodes in patients with cardiac resynchronization therapy	Prospective observational study	In 108 consecutive CRT patients (mean follow-up 12.6±4.9 months), AHRE (≥6 min, atrial rate >190 bpm) occurred in 31 (28.7%). Coronary-sinus galectin-3 was higher with AHRE (18.09±2.62 vs 13.17±3.17; p<0.001) and correlated with AHRE time burden (r=0.436; p<0.001); multivariable logistic regression retained LA volume (OR 1.127; 95% CI 1.045–1.216; p=0.002) and CS galectin-3 (OR 1.799; 95% CI 1.388–2.330; p<0.001) as independent predictors.

Simu GR, 2023 [63]	Galectin-3, Inflammation, and the Risk of Atrial High-Rate Episodes in Patients with Dual Chamber Pacemakers	Prospective observational study	In 102 dual-chamber pacemaker patients assessed at 1 year, AHRE incidence was 67%, with higher baseline galectin-3 in those with AHRE. A galectin-3 threshold >990 pg/mL showed moderate discrimination (AUC 0.63; 95% CI 0.52–0.73; p=0.04) and was associated with AHRE in univariate analysis (OR 1.0012; 95% CI 1.0001–1.0023; p=0.0328), but multivariable models identified LAVI as the only independent predictor (OR 1.0883; 95% CI 1.0351–1.1441; p=0.0009).
Liao M-T, 2020 [64]	High-Sensitivity C-Reactive Protein is a Predictor of Subsequent Atrial High-Rate Episodes in Patients with Pacemakers and Preserved Ejection Fraction	Prospective observational study	In 171 pacemaker recipients with preserved EF followed for a median of 614 days, 66 (39%) developed subsequent AHRE. In multivariable Cox regression, hs-CRP independently predicted AHRE (HR 1.121; 95% CI 1.015–1.238; p=0.024), supporting an inflammation-linked signal for incident device-detected atrial tachyarrhythmias in this cohort.
Satilmis S, 2018 [65]	Role of the monocyte-to-high-density lipoprotein ratio in predicting atrial high-rate episodes detected by cardiac implantable electronic devices	Prospective observational study	In 203 sick-sinus-syndrome patients implanted with a dual pacemaker, interrogation at 6 months identified AHRE in 51 (25.1%). The monocyte-to-HDL (M/H) ratio was higher with AHRE (11.41±1.24 vs 8.17±1.02; p<0.01) and showed a strong independent association with AHRE occurrence in multivariable analysis (OR 22.813; 95% CI 6.852–75.953; p<0.01).
Ji L, 2023 [66]	Monocyte/High-Density Lipoprotein Ratio Is Associated with Atrial High-Rate Episodes within One Year Detected by Cardiac Implantable Electronic Devices	Retrospective observational study	In 140 dual-chamber pacemaker recipients, 27 developed AHRE within 1 year (incidence 19.29%), and 44 developed AHRE over the entire follow-up. After multivariable adjustment, only M/H ratio ≥4.5 (vs <4.5) remained significant, with an adjusted HR of 4.313 (95% CI 1.675–11.105).
Wakula P, 2017 [67]	CHA$_2$DS$_2$-VASc score and blood biomarkers to identify patients with atrial high-rate episodes and paroxysmal atrial fibrillation	Case-control study	In 93 device patients (49 with AHRE; 44 without), several circulating biomarkers differed between groups, with the best discriminatory performance reported for TIMP-4. NT-proBNP >150 pg/mL was strongly linked to AHRE (OR 12.9), and combining two biomarkers further improved AHRE discrimination; overall, TIMP-4, NT-proANP, and NT-proBNP showed the strongest associations with both AHRE and paroxysmal AF, improving risk stratification beyond CHA2DS2-VASc alone.
Diederichsen SZ, 2020 [68]	Incidence and predictors of atrial fibrillation episodes as detected by implantable loop recorder in patients at risk: From the LOOP study	Secondary/post hoc analysis of an RCT cohort	In the LOOP ILR-screened at-risk cohort, 3-year cumulative incidence of AF was 33.8% for episodes ≥6 min, 16.1% for ≥5.5 h, and 5.7% for ≥24 h. Slower resting sinus rate, higher BMI, and higher baseline NT-proBNP and troponin T were independently associated with AF detection, and adding these markers to age/sex/comorbidity models improved discrimination for longer AF episodes (AUC 79% vs 65%, P=0.037).

Hnatek T, 2016 [69]	Factors underlying elevated troponin I levels following pacemaker primo-implantation	Prospective observational study	In 219 consecutive pacemaker primo-implantations, troponin I rose from 0.03±0.07 µg/L at baseline to 0.09±0.18 µg/L at 6 h and remained elevated at 18 h, consistent with frequent transient biomarker release after implantation. Greater troponin increase correlated with fluoroscopy (skiascopy) duration (P<0.001) and with lead fixation/penetration characteristics (P<0.001).
Turer AT, 2011 [70]	Myocardial ischemia induced by rapid atrial pacing causes troponin T release detectable by a highly sensitive assay: insights from a coronary sinus sampling study	Prospective observational study	During rapid atrial pacing, hs-cTnT increased in coronary sinus blood from 6.8 to 15.6 pg/mL (P<0.0001) and was mirrored later in peripheral blood (5.1 to 11.8 pg/mL at 180 min, P<0.0001). Absolute/relative troponin changes were similar across ischemia-stratified groups (e.g., absolute change 6.8 vs 8.8 pg/mL, P=0.50), supporting measurable troponin release even when objective ischemia signals are not clearly different.
Zecchin M, 2020 [71]	Atrial signal amplitude predicts atrial high-rate episodes in implantable cardioverter defibrillator patients: Insights from a large database of remote monitoring transmissions	Retrospective observational study	In a remote-monitoring cohort of 2976 ICD/CRT-D patients, baseline atrial sensing amplitude was lower among those who developed ≥24 h AHRE; among sensing-capable devices, 20.0% developed 24 h AHRE (event rate 9.3/100 patient-years). Atrial sensing >1.5 mV was associated with substantially lower 24 h AHRE risk (adjusted HR 0.52; 95% CI 0.33–0.83; P=0.006).
Biffi M, 2024 [72]	Device-detected atrial sensing amplitudes as a marker of increased risk for new onset and progression of atrial high-rate episodes	Retrospective remote-monitoring observational study	In a large CIED cohort, lower device-detected P-wave amplitude was associated with higher AHRE risk across clinically relevant thresholds (from ≥15 min to ≥7 days) independent of CHA2DS2-VASc. P-wave amplitudes showed a linear decline in the year preceding first AHRE (−7.3%; 95% CI 5.1–9.5; P<0.001), and amplitudes <2.45 mV identified patients at higher risk of AHRE onset and progression.
Chen Y, 2024 [73]	Is conduction system pacing more effective than right ventricular pacing in reducing atrial high-rate episodes in patients with heart failure and preserved ejection fraction?	Retrospective observational study	In patients without prior AF, conduction system pacing (CSP) was linked to fewer new-onset AHRE than right ventricular pacing (RVP), with the clearest separation when ventricular pacing was ≥20% (9.52% vs 29.70%, P=0.001). After adjustment, CSP remained associated with lower AHRE risk (HR 0.336; 95% CI 0.142–0.795; P=0.013), while larger LA diameter predicted AHRE (HR 1.109; 95% CI 1.048–1.173; P<0.001); in those with prior AF, AHRE progression did not differ significantly between CSP and RVP.
Yang W-Y, 2024 [74]	Comparison between left bundle branch area pacing and right ventricular pacing: ventricular electromechanical synchrony and risk of atrial high-rate episodes	Retrospective observational study	Over follow-up, new AHREs occurred in 12.5% of LBBAP patients versus 36.4% with RVA pacing and 61.5% with RVS pacing. LBBAP showed lower AHRE risk compared with pooled RVP (log-rank P=0.043) and compared with RVS specifically (log-rank P=0.027).

Zhang S, 2024 [75]	New-onset atrial high-rate episodes in left bundle branch area pacing versus right ventricular pacing for patients with atrioventricular block	Prospective observational study	In atrioventricular block patients without prior AF, LBBAP was associated with fewer new-onset AHRE than RVP; after propensity matching, AHRE incidence remained lower with LBBAP (11.6% vs 32.6%, P=0.02). Time-to-event analysis showed longer AHRE-free survival with LBBAP (HR 0.274; 95% CI 0.113–0.692; P=0.007).
Pestrea C, 2025 [76]	Mid-term comparison of new-onset AHRE between His bundle and left bundle branch area pacing in patients with AV block	Prospective observational study	In AV block patients, new-onset AHRE occurred in 8/59 (13.5%) with His-bundle pacing and 14/83 (16.8%) with LBBAP, with no significant difference in AHRE-free survival (HR 0.91; log-rank P=0.84). Most detected AHREs were low-burden overall (typically <1%).
Van Gelder IC, 2017 [77]	Duration of device-detected subclinical atrial fibrillation and occurrence of stroke in ASSERT	Secondary/post hoc analysis of an RCT cohort	In ASSERT, SCAF >24 h was associated with a significantly higher risk of ischemic stroke/systemic embolism (HR 3.24; 95% CI 1.51–6.95; P=0.003). Follow-up time after shorter SCAF durations (>6 min–6 h and >6–24 h) was not associated with increased risk versus periods without SCAF, supporting duration-dependent risk escalation.
Healey Jeff S, 2024 [78]	Apixaban for Stroke Prevention in Subclinical Atrial Fibrillation	Randomized controlled trial (RCT)	In ARTESiA (apixaban vs. aspirin), participants had a mean CHA_2DS_2-VASc score of 3.9±1.1, and the median duration of the longest SCAF episode within the 6 months before enrollment was 1.47 hours; apixaban reduced stroke or systemic embolism but increased major bleeding.
Kirchhof P, 2023 [79]	Anticoagulation with Edoxaban in Patients with Atrial High-Rate Episodes	Randomized controlled trial (RCT)	In NOAH-AFNET 6 (edoxaban vs. placebo), patients had a median CHA_2DS_2-VASc score of 4 and typically short episodes, with median longest AHRE of 2.8 hours; edoxaban did not significantly reduce the composite endpoint of cardiovascular death, stroke, or systemic embolism and increased major bleeding, with overall stroke rates remaining low.

Summary of included studies related to AHRE/SCAF. AAI-DDD, AAI/DDD mode switching; AF, atrial fibrillation; AHI, apnea–hypopnea index; AHRE, atrial high-rate episode(s); ARTESiA, Apixaban for the Reduction of Thrombo-Embolism in Patients With Device-Detected Subclinical AF; ASSERT, Asymptomatic AF and Stroke Evaluation in Pacemaker Patients and the AF Reduction Atrial Pacing Trial; AT, atrial tachycardia/tachyarrhythmia; AUC, area under the curve; C_2HEST, C_2HEST score; CHA_2DS_2-VASc, CHA_2DS_2-VASc score; $CHADS_2$, $CHADS_2$ score; CI, confidence interval; CIED, cardiac implantable electronic device(s); CMR, cardiac magnetic resonance; CRT, cardiac resynchronization therapy; CRT-D, cardiac resynchronization therapy defibrillator; CSP, conduction system pacing; CT, computed tomography; DDAF, device-detected atrial fibrillation; DDDR, dual-chamber rate-responsive pacing; DSC, Dice similarity coefficient; DWS, diastolic wall strain; ECG, electrocardiogram; ESUS, embolic stroke of undetermined source; GLS, (LV) global longitudinal strain; HATCH, HATCH score; HAT_2CH_2, HAT_2CH_2 score; HeartLogic, HeartLogic HF diagnostic algorithm; HF, heart failure; HFrEF, heart failure with reduced ejection fraction; HR, hazard ratio; hs-CRP, high-sensitivity C-reactive protein; hs-cTnT, high-sensitivity cardiac troponin T; IAB, interatrial block; ICC, intraclass correlation coefficient; ICD, implantable cardioverter-defibrillator; ILR,

implantable loop recorder; IN-alert, HeartLogic index alert status; IRR, incidence rate ratio; LA, left atrium/left atrial; LAA, left atrial appendage; LACI, left atrial coupling index; LAEF, left atrial emptying fraction; LASct, left atrial contraction strain; LAVI, left atrial volume index; LAVImax, maximum left atrial volume index; LAVmax/LAVmin, maximal/minimal left atrial volume; LBBAP, left bundle branch area pacing; LGE, late gadolinium enhancement; LOOP, LOOP study; LV, left ventricle/left ventricular; LVEDD, left ventricular end-diastolic diameter; LVEF, left ventricular ejection fraction; MACCE, major adverse cardio/cerebrovascular events; MACE, major adverse cardiovascular events; M/H, monocyte-to-HDL cholesterol ratio; MVP, MVP score (as defined by the original authors); NOAH-AFNET 6, Non-vitamin K Antagonist Oral Anticoagulants in Patients With AHREs trial; NT-proANP, N-terminal pro–atrial natriuretic peptide; NT-proBNP, N-terminal pro–B-type natriuretic peptide; OR, odds ratio; OSA, obstructive sleep apnea; PAC, premature atrial contraction(s); PALS/PACS, peak atrial longitudinal strain/peak atrial contraction strain; PWDis, P-wave dispersion; PWPT, P-wave peak time; PWPTV1/PWPTD2, P-wave peak time in lead V1/lead II; RDI, respiratory disturbance index; ROC, receiver operating characteristic; RVA/RVP/RVS, right ventricular apical pacing/right ventricular pacing/right ventricular septal pacing; SCAF, subclinical atrial fibrillation; SVT, supraventricular tachycardia; TIMP-4, tissue inhibitor of metalloproteinases-4; TSH, thyroid-stimulating hormone.
